# Selinexor for the treatment of recurrent or metastatic salivary gland tumors: Results from the GEMS‐001 clinical trial

**DOI:** 10.1002/cam4.6589

**Published:** 2023-10-11

**Authors:** Alberto Hernando‐Calvo, Eoghan Malone, Daphne Day, Amy Prawira, Ilan Weinreb, S. Y. Cindy Yang, Horace Wong, Angela Rodriguez, Sarah Jennings, Anneli Eliason, Lisa Wang, Anna Spreafico, Lillian L. Siu, Aaron R. Hansen

**Affiliations:** ^1^ Division of Medical Oncology and Hematology, Princess Margaret Cancer Centre. Department of Medicine University of Toronto Toronto Ontario Canada; ^2^ Princess Margaret Cancer Centre Toronto Ontario Canada

**Keywords:** biomarkers, drug design, experimental therapeutics, head and neck cancer, next generation sequencing, salivary gland tumors

## Abstract

**Objectives:**

We aimed to evaluate the activity of selinexor, an oral selective inhibitor of nuclear export, in patients with recurrent or metastatic salivary gland tumors (SGT).

**Methods:**

GEMS‐001 is an open‐label Phase 2 study for patients with recurrent or metastatic SGT with two parts. In Part 1 of the protocol, patients had tumor samples profiled with targeted next generation sequencing as well as immunohistochemistry for androgen receptor, HER‐2 and ALK. For Part 2, patients with no targeted therapies available were eligible to receive selinexor 60 mg given twice weekly every 28 days. The primary endpoint was objective response rate. Secondary endpoints included progression‐free survival (PFS) and prevalence of druggable alterations across SGT.

**Results:**

One hundred patients were enrolled in GEMS‐001 and underwent genomic and immunohistochemistry profiling. A total of 21 patients who lacked available matched therapies were treated with selinexor. SGT subtypes (WHO classification) included adenoid cystic carcinoma (*n* = 10), salivary duct carcinoma (*n* = 3), acinic cell carcinoma (*n* = 2), myoepithelial carcinoma (*n* = 2), carcinoma ex pleomorphic adenoma (*n* = 2) and other (n = 2). Of 18 evaluable patients, stable disease (SD) was observed in 17 patients (94%) (SD ≥6 months in 7 patients (39%)). However, no objective responses were observed. The median PFS was 4.9 months (95% confidence interval, 3.4–10). The most common treatment‐related Grade 1–2 adverse events were nausea [17 patients (81%)], fatigue [16 patients (76%)], and dysgeusia [12 patients (57%)]. Most common treatment‐related Grade 3–4 adverse events were hyponatremia [3 patients (14%)], neutrophil count decrease [3 patients (14%)] and cataracts [2 patients (10%)]. No treatment‐related deaths were observed.

**Conclusions:**

Although tumor reduction was observed across participants, single agent selinexor anti‐tumor activity was limited.

## 1 INTRODUCTION

Malignant salivary gland tumors (SGT) are rare diseases and constitute less than 1% of all cancers, with an annual incidence of approximately 1 case/100,000 worldwide.^1^ Salivary gland tumors differ in etiological, clinical, pathological, and genetic features when compared to head and neck squamous cell carcinomas.^2^ According to the World Health Organization (WHO) classification, there are different subtypes of SGT with different molecular and prognostic implications.^3^ Additionally, SGT encompass a heterogeneous group of diseases arising from different locations including major salivary glands (parotid, submandibular, and sublingual) and minor salivary glands.^4^ Overall, the prognosis of patients with recurrent inoperable or metastatic SGT remains limited.^5^

Notably, substantial clinical variability is observed across SGT subtypes ranging from an indolent growth pattern for a subset of individuals, while others have more aggressive disease with limited survival outcomes.^6^ Currently, NCCN guidelines recommend determination of androgen receptor (AR) and HER‐2 overexpression for metastatic SGT patients to guide molecularly targeted therapies in case of positivity.^7^ The assessment of *NTRK* fusions is also recommended for patients with mammary analogue secretory carcinoma SGT subtype. Despite these advances, the vast majority of SGT lack effective systemic options, with no health authority‐approved agents for a specific SGT indication. Recently, anti‐VEGF therapies have shown some success in patients with adenoid cystic carcinoma subtype with a limited disease control.^8^, ^9^ Hence, there is an unmet need to develop new therapeutic options for these orphan diseases.

Both tumor suppressor proteins (TSP) and growth regulatory proteins (GRP) utilize a single non‐redundant nuclear export protein complex in order to exit the nucleus. Exportin 1 (XPO1), also referred to as chromosomal region maintenance protein 1 (CRM1), is the primary component of this export complex and is overexpressed in many types of cancers.^10^, ^11^, ^12^, ^13^, ^14^ XPO1 is known to eliminate the function of TSP by exporting multiple of such proteins out of the nucleus, including but not limited to RB1, p53, BRCA1, and p27.^15^ Genomic alterations in pathways regulated by XPO1 have been involved in SGT carcinogenesis and progression.^16^, ^17^ Selinexor (KPT330) is a selective inhibitor of nuclear export (SINE) that slowly reversibly binds to and inactivates XPO1, thereby forcing the nuclear retention and functional activation of key TSP/GRP. Transient retention of TSP/GRP in the nucleus at high levels via XPO1 blockade activates cell cycle checkpoints and genome surveying actions.^13^

Considering the paucity of systemic therapies available for SGT, we hypothesized that treatment with selinexor may be able to elicit anti‐cancer responses. Here we present the results of the molecularly unmatched cohort of the GEMS‐001 trial investigating selinexor for recurrent unresectable or metastatic SGT patients with no actionable alterations or matched therapies available.

## 2 METHODS

### 2.1 Study design and patients

**FIGURE 1:**
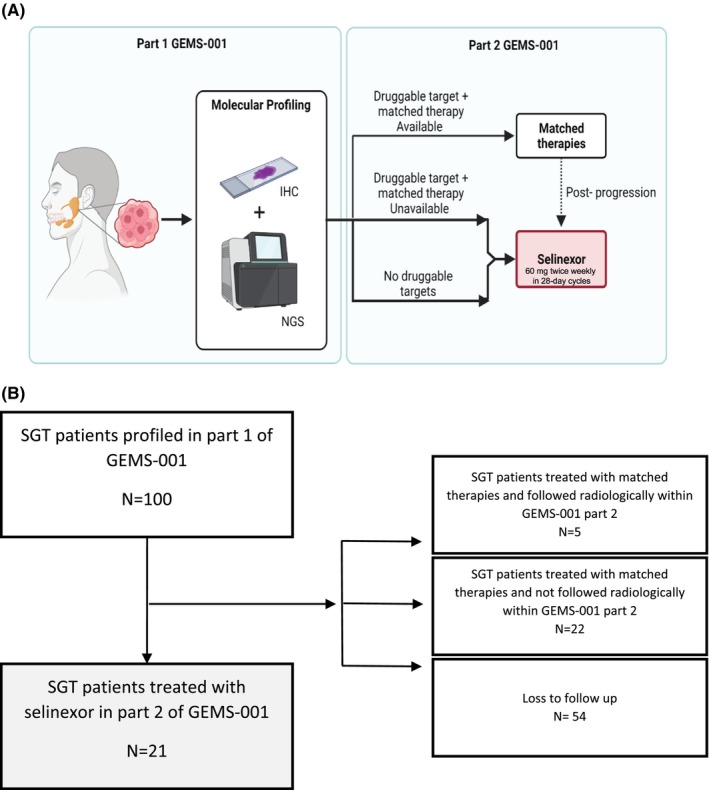
(A) Study design. Selinexor treatment was given oral, 60 mg given twice weekly (on Days 1 and 3 of each week) in 28‐day cycles. (B) GEMS‐001 flow diagram. IHC, immunohistochemistry; NGS, next generation sequencing; SGT, salivary gland tumors.

GEMS‐001 is an open‐label Phase 2 study for patients with recurrent or metastatic SGT with two parts. In Part 1 of the protocol, patients had tumor samples profiled with targeted next generation sequencing (NGS) as well as immunohistochemistry (IHC) for AR, HER‐2 and ALK. The molecular profiling data gathered during Part 1 of GEMS‐001 could be used to select patients for matched therapies within clinical trials available or via off‐label medications per compassionate use. For Part 2, patients with no targetable alterations identified or no matched agents available or having documented progression to all available matched agents, received selinexor 60 mg given twice weekly every 28 days. Patients could be treated with selinexor after progression to matched therapies available (Figure 1A).

Eligible patients for GEMS‐001 were ≥ 18 years old with an ECOG performance status of 0–1 and pathologically confirmed recurrent or metastatic SGT defined by WHO subtypes. Patients required evidence of clinical or radiological progression by RECIST version 1.1 assessed by the investigator at the time of study treatment in order to avoid enrollment of subjects with indolent disease. Additionally, patients required available archival (frozen or Formalin‐fixed paraffin‐embedded (FFPE)) tumor tissue or a fresh tumor specimen for molecular profiling. Patients also required measurable disease by RECIST 1.1 and adequate organ functions.

Specifically, to receive selinexor during Part 2, patients needed to have successful molecular profiling results with no actionable alterations or, if they had actionable alterations, no access to potential matched therapies. Patients had to be able to swallow oral medications with no evidence of bowel obstruction or infectious/inflammatory bowel disease. Prior anti‐cancer therapies received within 4 weeks before first cycle of selinexor or patients with active brain metastases were not allowed. There were no limits with respect of prior number of systemic treatment lines. Patients could not have a history of serious cardiac illness to be eligible for treatment with selinexor (see Text S1 for full inclusion and exclusion criteria). The study protocol was approved by Princess Margaret Cancer Centre ethics review board and all participants provided informed consent. Clinical trial is registered at ClinicalTrials.gov (NCT02069730).

### 2.2 Genomic and immunohistochemistry analyses

Both IHC and genomic analysis were performed with the use of a tumor‐tissue sample, preferably a sample obtained during the most recent progression. Patients enrolled underwent comprehensive IHC analysis including HER‐2, AR, and ALK and fluorescence in situ hybridization (FISH) in case HER‐2 IHC was 2+ according to current guidelines.^7^ NGS molecular profiling was performed utilizing different in‐house targeted panels including Sequenom, Illumina MiSeq next‐generation sequencing, TruSeq Amplicon Cancer Panel, 555Panel Hi5, and Oncomine panels (Text S2).

### 2.3 Treatment and assessments

In Part 2, the initial dose of selinexor was 60 mg given twice weekly (on Days 1 and 3 of each week) in 28‐day cycles. Pre‐specified dose modifications were defined in the protocol. The selected dose corresponds to the proposed recommended Phase 2 dose based on the Phase 1 dose escalation clinical trial testing selinexor in patients with all solid tumors.^18^ Patients received treatment until disease progression, unacceptable toxicity, withdrawal of consent, loss to follow‐up, or death. Imaging assessment was performed within 28 days before cycle 1 Day 1 (screening period), every 8 weeks for the first 6 months, and then every 12 weeks until disease progression or withdrawal for any other reason. Hematologic and biochemical laboratory tests were performed at screening, every 2 weeks for the first 8 weeks, and then every 4 weeks. Adverse events (assessed according to the National Cancer Institute Common Terminology Criteria, version 4.0) were recorded continuously until 30 days after the last dose of trial treatment.

### 2.4 Objectives and endpoints

The primary objective of GEMS‐001 was to assess the efficacy of selinexor in patients with recurrent or metastatic SGT as measured by objective response rate (ORR) by RECIST 1.1. Secondary objectives included progression‐free survival (PFS) assessed by the investigator and to analyze the genomic and molecular landscape of recurrent unresectable or metastatic SGT. PFS was defined as time from treatment initiation to disease progression or death from any cause.

### 2.5 Statistical analysis

Descriptive statistics were used to summarize all patient characteristics, treatment administration, and compliance efficacy end points and safety parameters. The Kaplan–Meier method was used to estimate PFS. If after enrollment of the first 18 patients evaluable by RECIST 1.1 no responses were observed, then no further accrual would occur in this phase. This study included a matched cohort arm for patients treated with molecularly targeted agents that has been reported separately and a molecularly unmatched cohort arm investigating selinexor reported here.^19^ However, this study was not designed to statistically compare the efficacy between the group of patients receiving selinexor versus matched therapies.

## 3 RESULTS

### 3.1 Baseline characteristics

Between July 2014 and September 2021, a total of 100 patients were enrolled in GEMS‐001 and underwent genomic and immunohistochemistry profiling in part 1. Of these, a total of 21 patients were treated with selinexor in Part 2 (Figure 1B). Among the patients that received treatment with selinexor, 12 patients were female and 9 patients male with a median age of 61 years (range 36–79). Salivary gland tumor subtypes (WHO classification) included adenoid cystic carcinoma (*n* = 10), salivary duct carcinoma (*n* = 3), acinic cell carcinoma (*n* = 2), myoepithelial carcinoma (*n* = 2), carcinoma ex pleomorphic adenoma (*n* = 2), poorly differentiated carcinoma (*n* = 1) and mucoepidermoid carcinoma (*n* = 1). Eight patients had SGT with a primary tumor located in a minor salivary gland while 13 had tumors arising from major salivary glands (9 patients from the parotid, 3 submandibular gland and 1 sublingual gland). Fourteen patients were treatment naïve and seven patients had received one or more lines of treatment prior to enrollment. Baseline characteristics of the patients treated with selinexor are shown on Table 1.

**TABLE 1:** Baseline characteristics of the overall population.

Characteristic	No. (%)
Age	
Median	61 (range 36–79)
Sex	
Male	9 (43%)
Female	12 (57%)
Race	
White	18 (86%)
Asian	2 (9%)
Unknown	1 (5%)
Ethnicity	
Non‐hispanic	19 (90%)
Hispanic or latino	1 (5%)
Unknown	1 (5%)
ECOG performance status	
0	7 (33%)
1	13 (62%)
Unknown	1 (5%)
Histology	
ACC	10 (48%)
SDC	3 (14%)
Other	8 (38%)
Tumor location	
Major salivary gland	13 (62%)
Minor salivary gland	8 (38%)
Prior treatment	
Radiation	21 (100%)
Surgery	20 (95%)
Number of prior lines	
0	14 (67%)
1	7 (33%)
NGS panel	
Sequenom	1 (5%)
TSACP	9 (43%)
555 panel. HI5	5 (24%)
Oncomine	6 (28%)
IHC results	
Present	20 (95%)
Unknown	1 (5%)
Actionable alterations	
Yes	8 (38%)
No	13 (62%)

Abbreviations: ACC, adenoid cystic carcinoma; IHC, Immunohistochemistry; SDC, salivary duct carcinoma; NGS, Next generation sequencing.

### 3.2 Genomic and molecular profiling of the whole population and selinexor cohort

**FIGURE 2:**
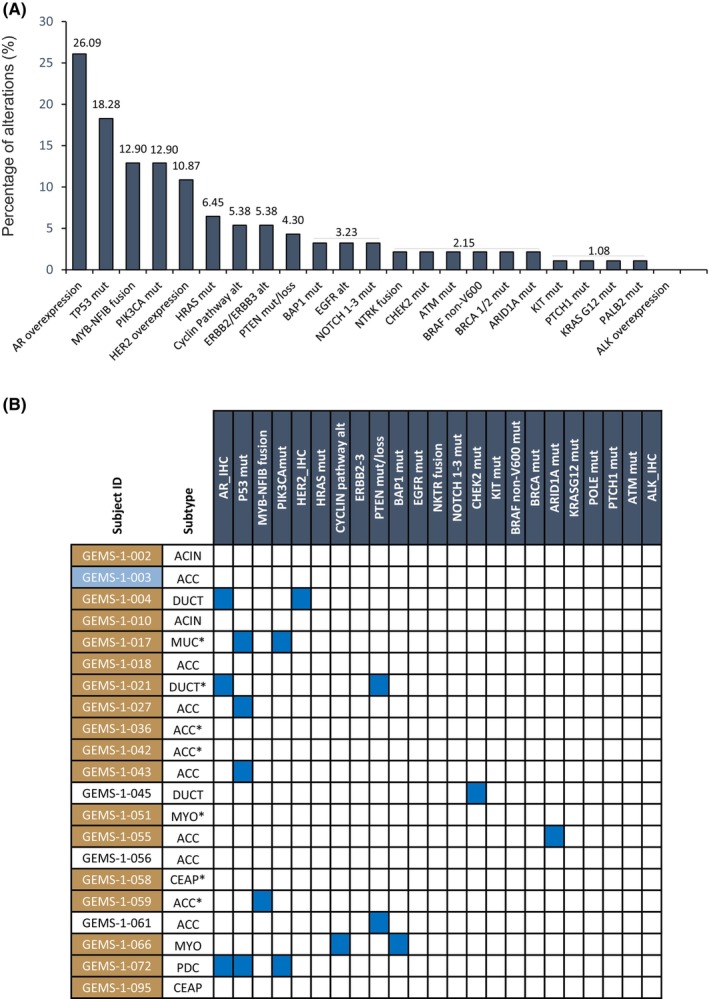
(A) Genomic and immunohistochemistry alterations observed in the part 1 of the GEMS‐001 protocol (*N* = 100). The number above the bar represents the percentage of the study population positive for each specific marker. (B) Genomic and immunohistochemistry alterations observed in patients receiving selinexor in the part 2 of GEMS‐001 protocol (*N* = 21). Subject ID identified with brown color achieved stable disease as best response and subject identified with blue color achieved progressive disease as best response. Subjects not identified with colors were not RECIST 1.1 evaluable. Alt, alteration; IHC, Immunohistochemistry; Mut, mutation; ACIN, acinic cell carcinoma; ACC, Adenoid cystic carcinoma; DUCT, Salivary duct carcinoma; ACIN, Acinic cell carcinoma; MUC, Mucoepidermoid carcinoma; MYO, Myoepithelial carcinoma; CEAP, Carcinoma ex pleomorphic adenoma; PDC, Poorly differentiated carcinoma. * SD ≥6 months.

Among the 100 patients enrolled in GEMS‐001, IHC AR overexpression was present in 26%, HER‐2 overexpression was present in 11% and ALK 0%. On NGS, *PIK3CA* mutations were present in 13%, *HRAS* mutations 6%, *ERBB2*/*3* alterations 5%, *NOTCH1*‐*3* mutations 3% and *ETV6*‐*NTRK3* fusion 2%. Up to 45% patients displayed at least 1 actionable alteration and 25% had 2 or more. Median number of actionable alterations were 1 (range 0–4). Actionable alterations were enriched in salivary duct carcinoma (94% ≥1 actionable alterations) as compared to acinic cell carcinoma (56% ≥1 actionable alterations), ACC (29% ≥1 actionable alterations) or other histologies (63% ≥1 actionable alterations) (*p* < 0.001 Fisher's exact test). Importantly, oncogenic or likely oncogenic mutations involved in DNA repair pathways (*CHEK2*, *ATM*, *BRCA1*/*2*, *ARID1A*, and *PALB2*) were present in 13% of patients (Figure 2A). Among the population exposed to selinexor, IHC analyses showed AR overexpression in three patients and HER‐2 overexpression in one patient. Targeted panel genomic profiling showed *PIK3CA* mutations in two patients, *PTEN* mutations or loss in two patients and *BAP1* in one patient. The most common genomic and IHC alterations observed in the overall cohort of GEMS‐001 and in the population treated with selinexor are shown on Figure 2A,B.

### 3.3 Efficacy outcomes

**FIGURE 3:**
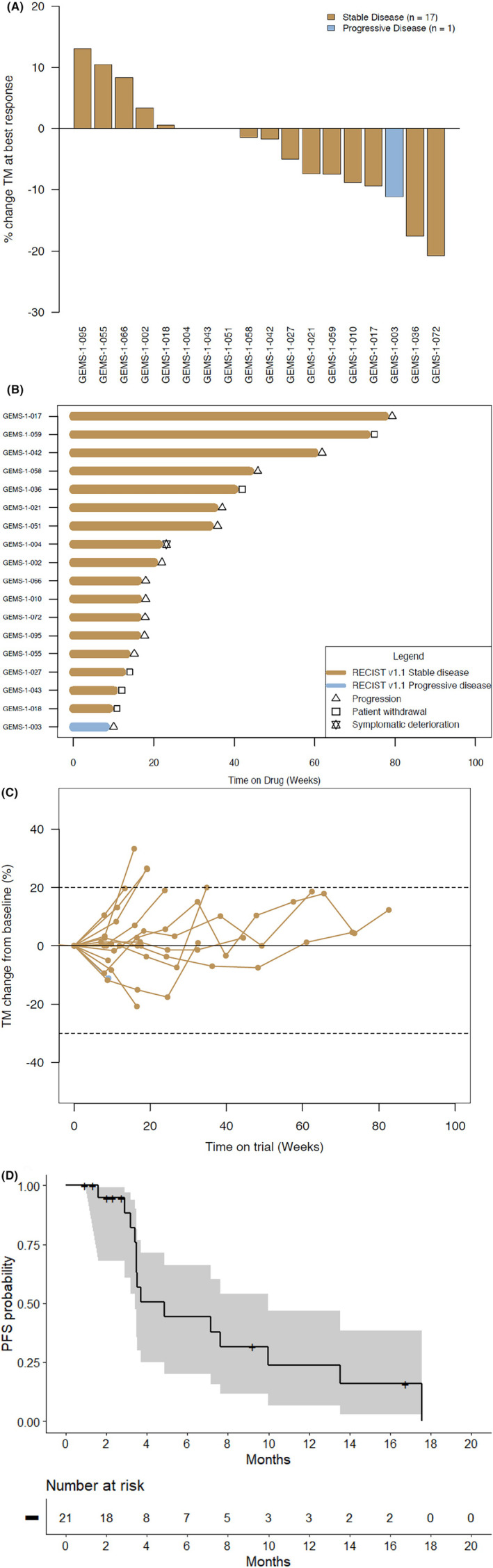
(A) Waterfall plot showing depth of response of the target lesions (RECIST 1.1) among the evaluable population. Patient with progressive disease as best response had tumor reduction on target lesions but new lesions were recorded. (B) Swimmer plot showing duration on treatment and reason for treatment discontinuation. (C) Spider plot showing the evolution of the sum of the target lesions during treatment. (D) Progression free survival (PFS) analysis: Median PFS time was estimated to be 4.9 95% confidence interval (3.4–10.0) months. The gray lines indicate the 95% confidence interval. TM, target lesions measurements.

Within the cohort of 21 patients treated with selinexor, 3 were not RECIST 1.1 evaluable for response due to insufficient duration on treatment, 2 patients due to poor tolerability and 1 withdrew consent due to other reasons. Among the RECIST 1.1 evaluable population (*n* = 18), the median number of cycles of selinexor received were 4 (range: 1–19). Stable disease (SD) as best response was observed in 17 patients (94%) (SD ≥6 months in 7 patients [39%]) (Table 2). Four patients in the cohort had *TP53* mutant tumors. All of them had SD as best response. Progressive disease as best response was observed in one patient (6%), adenoid cystic carcinoma. Tumor reduction of target lesions was observed in 11 patients (61%). However, no partial or complete responses were observed. The median PFS was 4.9 months (95% confidence interval, 3.4–10 months) (Figure 3). No association was observed between patients achieving SD ≥6 months and genomic or IHC alterations (Figure 2B). A total of 12 patients discontinued selinexor due to progression, five withdrew consent due to poor tolerability to selinexor and 1 due to symptomatic progression (Table 2).

**TABLE 2:** Distribution of responses and reasons for treatment discontinuation in the RECIST 1.1 evaluable population.

Best overall response	No. (%)
Complete/partial response	0 (0)
Stable disease	17 (94)
Progression of disease	1 (6)
RECIST 1.1 non evaluable	3*(14)
Reason for discontinuation	
Continue on study	0 (0)
Withdrawal of consent	5 (28)
Progression of disease	12 (67)
Symptomatic progression	1 (6)

* Percentage includes RECIST 1.1 evaluable and non‐evaluable population as denominator.

### 3.4 Safety

The most common treatment‐related Grade 1–2 adverse events were nausea (17 patients [81%]), fatigue (16 patients [76%]), and dysgeusia (12 patients [57%]). Most common treatment‐related Grade 3–4 adverse events were hyponatremia (3 patients [14%]), neutrophil count decrease (3 patients [14%]) and cataracts (2 patients [10%]). No treatment‐related deaths were observed. More than half of the study population (62%) required one or more dose reductions of selinexor during treatment. Median number of dose reductions was 1 (range 0–2). The most frequent treatment‐related and treatment‐emergent adverse events present in ≥10% of the population are described in Table 3 and Table S1.

**TABLE 3:** Summary of treatment‐related adverse events occurring in ≥10% of the trial participants.

AE	No. (%)					
	Grade 1	Grade 2	Grade 3	Grade 4	Grade 5	Total
Gastrointestinal						
Nausea	12 (57.1)	5 (23.8)				17 (80.9)
Anorexia	4 (19.0)	7 (33.3)	1 (4.8)			12 (57.1)
Dysgeusia	9 (42.8)	3 (14.3)				12 (57.1)
Vomiting	8 (38.1)	1 (4.8)	1 (4.8)			10 (47.6)
Constipation	7 (33.3)					7 (33.3)
Dehydration		5 (23.8)				5 (23.8)
Diarrhea	1 (4.8)	2 (9.5)				3 (14.3)
Constitutional						
Fatigue	6 (28.6)	10 (47.6)	1 (4.8)			17 (80.9)
Weight loss	4 (19.0)	4 (19.0)	1 (4.8)			9 (42.8)
Generalized muscle weakness	1 (4.8)	3 (14.3)				4 (19.0)
Blood						
Neutrophil count decreased	1 (4.8)	5 (23.8)	2 (9.5)	1 (4.8)		9 (42.8)
Anemia		3 (14.3)				3 (14.3)
Metabolic or laboratory						
Hyponatremia	2 (9.5)		3 (14.3)			5 (23.8)
Creatinine increased	2 (9.5)	1 (4.8)				3 (14.3)
Neurological						
Dizziness	4 (19.0)	2 (9.5)				6 (28.6)
Headache	3 (14.3)					3 (14.3)
Vertigo	3 (14.3)					3 (14.3)
Other						
Blurred vision	9 (42.8)					9 (42.8)
Alopecia	4 (19.0)					4 (19.0)

## 4 DISCUSSION

We report on the activity of selinexor in patients with recurrent or metastatic SGT. Although tumor reduction was achieved across participants and the side effect profile was tolerable, no confirmed partial or complete responses were observed. Historically there are few effective systemic therapies for SGT. Chemotherapy for all SGT histologies and targeted therapies for a subset of patients are potential treatment options. Importantly, these therapies have shown benefit in a small subset of patients underscoring the necessity to advance drug development for these rare tumors.^9^, ^20^, ^21^, ^22^ Recently, the understanding of molecular aberrations in SGT has revealed promising targets for matched therapeutics which include NTRK inhibitors and HER‐2 or AR blockade. However, despite these advances, the majority of patients will not have actionable alterations on multigene panel testing and thus may not benefit from molecularly targeted approaches.^19^ Furthermore, the lack of access to clinical trials testing targeted therapies may also be a barrier for treatment. Consequently, there is an urgent need to identify treatments that are broadly effective in an unselected population of patients with SGT.

In our cohort, a total of 21 participants with no actionable alterations, or with actionable alterations but no targeted therapies available received treatment with selinexor single agent 60 mg twice weekly. Although no complete or partial responses were observed, more than half of the population achieved tumor reduction in the measurements of the target lesions. Moreover, more than one third of the population achieved prolonged disease stabilization over 6 months. From a safety perspective, the toxicity profile was consistent with already published data from patients with Diffuse Large B‐Cell Lymphoma or multiple myeloma, the two indications for which selinexor has received FDA approval.^23^, ^24^

The genomic and molecular profiles of the GEMS‐001 participants were consistent with already published data.^25^, ^26^ Despite comprehensive genomic and molecular profiling, no alterations were observed to correlate with selinexor efficacy probably because of the study design enrolling patients without targetable alterations to the selinexor treatment arm and the small study population. In our cohort, comprehensive molecular profiling unveiled actionable alterations in almost half of the population enrolled in GEMS‐001 Part 1. The breadth of druggable alterations inform potential pathways that may be targeted in conjunction to selinexor to maximize benefit. Specifically, DNA Damage Response and Repair (DDR) pathway alterations were observed in 13% of the enrolled population. Notably, selinexor has been shown to decrease the expression of DDR proteins sensitizing preclinical models to DNA damage agents.^27^, ^28^ Selinexor is being investigated in combinations with PARP‐inhibition (NCT05035745), chemotherapy agents (NCT02269293, NCT02384850, and NCT03555422) and radiotherapy (NCT05099003 and NCT04216329) in different solid tumors. Considering the limited therapeutic opportunities for recurrent or metastatic SGT and the variety of cellular pathways in which XPO1 is involved, combination treatment strategies may be required to improve anti‐tumor responses.

Despite the unique cohort of SGT patients with genomic and IHC profiling available enrolled in GEMS‐001, our study has limitations. Firstly, considering the target population treated with selinexor, only patients with no available matched therapies or no druggable alterations were selected. Whether the presence of actionable genomic alterations has prognostic implications is unknown. Moreover, GEMS‐001 was not designed to prospectively follow‐up participants ineligible for selinexor treatment. Hence, many patients were lost to follow‐up for prospective evaluation after genomic and IHC profiling. In our study, patients required tissue‐based genomic and IHC analyses prior to treatment initiation with selinexor. Presumably given that only patients who had progressing tumors were eligible to enroll onto selinexor and thus had an inherent poor prognosis which could have impacted the outcomes and moderate signs of activity observed in this cohort. The advancements in non‐invasive techniques for genomic analyses such as liquid biopsies may, in the near future, may help to inform in a timely manner treatment selection strategies.^29^, ^30^ Lastly, the small number of patients enrolled and the variety of SGT histologies included may have hindered the possibility to identify activity signals in specific subtypes of SGT or individuals with specific genomic alterations. Further multi‐institutional efforts or master protocols will be required to fully elucidate the role of selinexor in biomarker enriched populations of SGT and its role with other molecularly‐guided therapies.

In conclusion, although tumor reduction was observed across participants, single agent selinexor anti‐tumor activity was limited. The tumor reduction observed in more than half of the study participants suggest a further role for biomarker development and rational combination strategies.

## AUTHOR CONTRIBUTIONS

**Alberto Hernando‐Calvo:** Conceptualization (equal); data curation (equal); formal analysis (equal); investigation (equal); methodology (equal); project administration (equal); validation (equal); writing – original draft (equal); writing – review and editing (equal). **Eoghan Malone:** Investigation (equal); project administration (equal). **Daphne Day:** Supervision (equal); writing – review and editing (equal). **Amy Prawira:** Supervision (equal); writing – review and editing (equal). **Ilan Weinreb:** Supervision (equal); writing – review and editing (equal). **S.Y. Cindy Yang:** Writing – review and editing (equal). **Horace Wong:** Data curation (equal); formal analysis (equal); project administration (equal). **Angela Rodriguez:** Conceptualization (equal); project administration (equal). **Sarah Jennings:** Investigation (equal); project administration (equal). **Anneli Eliason:** Project administration (equal). **Lisa Wang:** Methodology (equal); software (equal); supervision (equal); visualization (equal); writing – review and editing (equal). **Anna Spreafico:** Conceptualization (equal); funding acquisition (equal); investigation (equal); methodology (equal); supervision (equal); writing – review and editing (equal). **Lillian L. Siu:** Funding acquisition (equal); investigation (equal); methodology (equal); project administration (equal); supervision (equal); writing – review and editing (equal). **Aaron R. Hansen:** Conceptualization (equal); formal analysis (equal); funding acquisition (equal); investigation (equal); methodology (equal); resources (equal); supervision (equal); writing – original draft (equal); writing – review and editing (equal).

## FUNDING INFORMATION

This study received partial funding by Karyopharm Therapeutics.

## CONFLICT OF INTEREST STATEMENT

Alberto Hernando‐Calvo has financial interests (personal, other, travel, accommodations, expenses) with Novartis, Merk Serono and Kyowa Kirin International. Eoghan Malone, Ilan Weinreb, S.Y. Cindy Yang, Horace Wong, Angela Rodriguez, Sarah Jennings, Anneli Eliason, and Lisa Wang report no conflicts of interest. Daphne Day has provided research support (clinical trials for institution): Beigene, Bristol‐Myers Squibb, EpimAb, Harbour BioMed, Maxinovel, MSD, Olema Pharmaceuticals, Pfizer, PhamAbcine, and Roche. Amy Prawira has provided funding to institutions for the conduct of clinical trials from Merck Serono Dohme, Bristol Myers Squibb, Astra Zeneca, Pfizer, Novartis, Beigene, Eli Lilly, Bayer, Deciphera, Boehringer Ingelheim, Amgen, PTC Therapeutics, Genfleet, Regeneron, and is the Director and Founder of Biointellix Pty Ltd. Anna Spreafico has the following financial relationships to disclose: Consultant for (Advisory Board): Merck (compensated), Bristol‐Myers Squibb (compensated), Oncorus (compensated), Janssen (compensated), Medison & Immunocore (compensated). Speaker's Bureau for: None. Grant/Research support from (Clinical Trials): Novartis, Bristol‐Myers Squibb, Symphogen AstraZeneca/Medimmune, Merck, Bayer, Surface Oncology, Northern Biologics, Janssen Oncology/Johnson & Johnson, Roche, Regeneron, Alkermes, Array Biopharma/Pfizer, GSK, Oncorus, Treadwell, Amgen. Stockholder in: None. Employee of: None. Lillian L. Siu has consulting/advisory arrangements with Merck, Pfizer, AstraZeneca, Roche, Symphogen, Seattle Genetics, GlaxoSmithKline, Voronoi, Arvinas, Tessa, Navire, Relay, Rubius, Janpix, Daiichi Sanyko, Coherus, Marengo, InteRNA; stock ownership of Agios (spouse); leadership position in Treadwell Therapeutics (spouse); and institution receives clinical trials support from Novartis, Bristol‐Myers Squibb, Pfizer, Boerhinger‐Ingelheim, GlaxoSmithKline, Roche/Genentech, Karyopharm, AstraZeneca, Merck, Celgene, Astellas, Bayer, Abbvie, Amgen, Symphogen, Intensity Therapeutics, Mirati Therapeutics, Shattucks. Aaron Hansen has obtained research funding from GSK, Merck, Pfizer, MedImmune/Genentech, Roche, Janssen, BMS, AstraZeneca, Astellas, Boehringer Ingelheim, and Bayer, and has played a consulting role and served on the advisory boards for GSK, Merck, and Eisai.

## ETHICS STATEMENT

This research project and the consent to participate were submitted and approved by the institutional review board of Princess Margaret Cancer Centre (CAPCR 13‐6955.29).

## CLINICAL TRIAL REGISTRATION

This clinical trial is registered at ClinicalTrials.gov (NCT02069730) registered February 20 2014.

## CONSENT FOR PUBLICATION

The investigators of the GEMS‐001 clinical trial consent the publication of this data as collected in the protocol.

## Supporting information


Data S1.

## Data Availability

The data generated in this study are available within the article and its supplementary data files. Data acquired and/or used in the study could be made available on reasonable requests.
