# Supplementary material for: Selinexor for the treatment of recurrent or metastatic salivary gland tumors: Results from the GEMS‐001 clinical trial
Source: Cancer Med. 2023 Oct 11;12(20):20299–310. doi: 10.1002/cam4.6589 (PMC10652322; doi:10.1002/cam4.6589)
Supplement: Supplementary file 1 — Data S1. [file CAM4-12-20299-s001.docx]

**SELINEXOR FOR THE TREATMENT OF RECURRENT OR METASTATIC SALIVARY GLAND TUMORS: RESULTS FROM THE GEMS-001 CLINICAL TRIAL**

**Title:** Selinexor for the treatment of recurrent or metastatic salivary gland tumors: results from the GEMS-001 clinical trial

**Authors:**

Alberto Hernando-Calvo^1^, Eoghan Malone^1^, Daphne Day^1^, Amy Prawira^1^, Ilan Weinreb^1^, S. Y. Cindy Yang^2^, Horace Wong^2^, Angela Rodriguez^2^, Sarah Jennings^2^, Anneli Eliason^2^, Lisa Wang^2^, Anna Spreafico^1^, Lillian L. Siu^1^, Aaron R. Hansen^1^

**Affiliations:**

1- Division of Medical Oncology and Hematology, Princess Margaret Cancer Centre. Department of Medicine, University of Toronto, Toronto, Ontario, Canada.

2- Princess Margaret Cancer Centre, Toronto, Ontario, Canada.

**Corresponding Author:**

**Aaron R. Hansen**

aaron.r.hansen@health.qld.gov.au

Princess Margaret Cancer Centre,

University Health Network,

University of Toronto,

Toronto, Canada

**SUPPLEMENTARY**

**Supplementary text 1.** Inclusion and exclusion criteria

The following eligibility criteria are designed to select patients for whom protocol treatment is considered appropriate. Patients will be recruited from a population of patients with recurrent of metastatic SGT referred to Princess Margaret Cancer Centre.

Enrollment into Phase 1 (molecular profiling phase) is mandatory for enrollment on the study as molecular profiling is a critical element of this study. However, enrollment into Phase 2 is neither required nor guaranteed by enrollment into the profiling phase. Patients must have a performance score of 0 to 1 to be enrolled into either phase of the study.

All relevant medical and non-medical conditions should be considered when deciding whether this protocol is suitable for a particular patient.

## Inclusion criteria

Each patient must meet all of the following criteria to be enrolled in this study:

Enrollment in the profiling phase (Phase 1) requires:

1. Patients must have available archival tumor tissue (Frozen or FFPE) or fresh tumor specimen from histological tissue for molecular profiling.
2. Histological or cytological proof of malignant salivary gland tumor.
3. ECOG performance score 0-1.
4. Documented evidence of recurrent or metastatic SGT.

Enrollment in the treatment phase (Phase 2) requires:

NOTE:

- If the matched treatment is in the context of another phase I trial, the eligibility criteria of the enrolled trial will be used instead of the criteria from this trial.
- If matched treatment is provided outside a clinical trial, the eligibility requirement and treatment decision will be as per the discretion of the treating physician.
- For - Unmatched treatment / Matched treatment with no accessibility or treatment unavailable / Cross over after Progression on Matched treatment (Selinexor arm) the following criteria must be met:

1. Molecular profiling results in the profiling phase of this study (which includes but is not limited to tumor DNA sequencing)
2. Advanced recurrent or metastatic salivary gland cancer for which no curative therapy exists
3. Evidence of clinical or radiological disease progression at the time of study treatment
4. Patients must have adequate hematological, liver, renal and cardiac functions:
   - - - 1. Absolute neutrophil count ≥1.0 x 10^9^/L
         2. Platelet count ≥100 x 10^9^/L
         3. Serum creatinine < 1.5 x upper limit of normal (ULN) or calculated creatinine clearance > 50 mL/min
         4. Serum bilirubin <1.25 x ULN
         5. AST/ALT <2.5 x ULN (<5 x ULN for patients with liver metastases)
         6. QTc interval < 480 ms
5. Patients cannot have any history of serious cardiac illness including (but not confined to):
   - - - 1. Previous (< 12 months) or active myocardial infarction
         2. Congestive cardiac failure (NYHA III or IV)
         3. Unstable angina pectoris
         4. Recent coronary artery bypass grafting <6 months
         5. Uncontrolled hypertension despite optimal therapy (systolic≥160 mmHg or diastolic ≥100 mmHg)
         6. Ventricular arrhythmia < 6 months
6. Patients cannot have any serious medical conditions that might be aggravated by treatment or limit compliance. This includes, but not limited to uncontrolled psychiatric disorders, serious infections, active peptic ulcer disease and bleeding diathesis
7. Patients with CNS metastases are permitted provided these are clinically stable as defined by >30 days from surgery and/or radiotherapy, and no current glucocorticoids
8. Patients must be able to take oral medication and have no evidence of bowel obstruction, infectious/inflammatory bowel disease
9. Patients cannot have any other active malignancy at any other site
10. Patients must be ≥ 18 years of age on day of signing informed consent
11. Patients must have measurable disease as defined by RECIST v1.1 criteria
12. Patients may not receive any other concurrent investigational agent

## Exclusion criteria

Exclusion criteria for molecular profiling (Phase 1)

1. Patient refuses to consent to the use of his/her primary or metastatic SGT tissue for molecular profiling.
2. The amount of tumor sample is inadequate for standard molecular profiling as assessed by Princess Margaret Cancer Centre pathologist.
3. Patient’s life expectancy less than three months.

Exclusion criteria for treatment phase (Phase 2)

1. Patients who had stopped the previous systemic treatment but showed no clinical or radiological evidence of disease progression.
2. Patients who have received the same drug treatment prior to the enrollment in to treatment phase (Phase 2).

## Contraception Requirements

Patients should not become pregnant or father a child while on this study because the study treatments in this study can affect an unborn baby. Women should not breastfeed a baby while on this study. It is important that patients understand the need to use birth control while on this study. Female patients of childbearing potential must agree to use two methods of contraception (one highly effective and one effective) and have a negative serum pregnancy test at Screening, and male patients must use an effective barrier method of contraception if sexually active with a female of childbearing potential.

Highly effective methods include:

- Hormonal contraceptives (e.g., combined oral contraceptives, patch, vaginal ring, injectables, and implants)
- Intrauterine device or intrauterine system
- Vasectomy or tubal ligation
- Effective methods include:
- Barrier methods of contraception (e.g., male condom, female condom, cervical cap, diaphragm, contraceptive sponge).

Notes:

- *No barrier method by itself achieves a highly effective standard of contraception*
- *The proper use of diaphragm or cervical cap includes use of spermicide and is considered one barrier method.*
- *The cervical cap and contraceptive sponge are less effective in parous women.*
- *The use of spermicide alone is not considered a suitable barrier method for contraception.*
- *When used consistently and correctly, “double barrier” methods of contraception (e.g., male condom with diaphragm, male condom with cervical cap) can be used as an effective alternative to the highly effective contraception methods described above.*
- *Male and female condoms should not be used together as they can tear or become damaged.*

Alternatively, the following fulfill the contraception requirements:

A sexual partner who is surgically sterilized or post-menopausal.

Total (true) abstinence (when this is in line with the preferred and usual lifestyle of the patient), is an acceptable method of contraception. NOTE: Periodic abstinence (e.g., calendar, ovulation, symptothermal, post-ovulation methods) and withdrawal are not acceptable methods of contraception. For both male and female patients, effective methods of contraception must be used throughout the study and for 3 months following the last dose of study treatment.

**Supplementary text 2.** Targeted panels used at Princess Margaret Cancer Centre.

The Sequenom Solid Tumor Panel at Princess Margaret Cancer Centre includes single nucleotide polymorphisms genotyping of 23 genes for 279 mutations (hotspots, substitutions and insertions/deletions). The Ilumina MiSeq TruSeq Amplicon Cancer Panel at PM includes targeted sequencing of 48 genes and 212 amplicons. The Hi5 panel at PM is a custom hybridization capture ngs panel of 555 cancer-related genes [UHN Hi5 panel (SureSelect: Agilent, Santa Clara, CA, U.S.A.)] sequenced on the NextSeq platform (Illumina, San Diego, CA, U.S.A.). The Oncomine panel at PM is a commercial 161- gene amplicon dna/rna panel (Oncomine Comprehensive Assay v3: ThermoFisher Scientific, Waltham, MA, U.S.A.) sequenced on the IonS5XL platform (ThermoFisher Scientific).

**Supplementary table 1:** Treatment emergent adverse events occurring in ≥ 10% of the trial participants.
